# Postoperative study of vital capacity and ventilation measurements following elective craniotomy

**DOI:** 10.1590/S1516-31802008000100003

**Published:** 2008-01-03

**Authors:** Luciana Carrupt Machado Sogame, Sonia Maria Faresin, Milena Carlos Vidotto, José Roberto Jardim

**Keywords:** Craniotomy, Vital capacity, Postoperative complications, Neurosurgery, Respiratory function tests, Craniotomia, Capacidade vital, Complicações pós-operatórias, Neurocirurgia, Testes de função respiratória

## Abstract

**CONTEXT AND OBJECTIVE:**

Changes in pulmonary function commonly occur after general surgery. The aims were to evaluate vital capacity, tidal volume and respiratory frequency among patients undergoing elective craniotomy and to determine possible correlations of these parameters with surgery duration and etiology for neurosurgery.

**DESIGN AND SETTING:**

Prospective, open study at a tertiary university hospital.

**METHODS:**

Twenty-six patients underwent elective craniotomy for aneurysm clipping (11) or tumor resection (15). Vital capacity (VC), tidal volume (TV), minute volume (VE) and respiratory rate were determined before the operation and on the first to fourth postoperative days.

**RESULTS:**

There were significant decreases of 25% in VC, 22% in TV and 12% in VE (p < 0.05) and no significant increase in respiratory frequency (5%) on the first postoperative day. VE returned to baseline on the second postoperative day and TV on the third postoperative day, while VC was 8% lower on the fourth postoperative day, compared with before the operation (p < 0.05). VC reduction was significantly greater in patients undergoing aneurysm clipping (43%) than in patients undergoing tumor resection (14%) when surgery duration was more than four hours (p < 0.05), with no significant change when surgery duration was less than four hours.

**CONCLUSION:**

Reductions in VC, TV and VE were observed during the postoperative period in patients undergoing aneurysm clipping or tumor resection. The reductions in VC and TV were greater in patients undergoing craniotomy due to aneurysm and with longer surgery duration.

## RESUMO

**CONTEXTO E OBJETIVO:**

Após cirurgia é comum ocorrerem alterações na função pulmonar. O objetivo foi avaliar a evolução da capacidade vital, do volume corrente e da freqüência respiratória em pacientes submetidos a craniotomia eletiva e determinar uma possível correlação dessas variáveis com o tempo de cirurgia e a causa da neurocirurgia.

**TIPO DE ESTUDO E LOCAL:**

Prospectivo aberto realizado em hospital universitário terciário.

**MÉTODOS:**

Vinte e seis pacientes submetidos a craniotomia eletiva para clipagem de aneurisma (11) ou ressecção de tumor (15). A capacidade vital (CV), o volume corrente (VC), o volume minuto (VM) e a freqüência respiratória (FR) foram determinados durante o período pré-operatório e do primeiro ao quarto dia do pós-operatório (PO).

**RESULTADOS:**

Houve uma redução significante de 25% no CV, 22% no VC e 12% no VM (p < 0,05) e aumento não significante da FR (5%) no primeiro dia de PO. O VM retornou ao normal no segundo PO e o VC no terceiro PO, enquanto a CV continuou 8% menor no quarto PO comparado com o período pré-operatório (p < 0,05). A redução na CV foi significantemente maior em pacientes submetidos a clipagem de aneurisma (43%) do que em pacientes submetidos a ressecção de tumor (14%) quando o tempo de cirurgia foi maior que quatro horas (p < 0,05), sem apresentar diferença quando o tempo cirúrgico foi menor que quatro horas.

**CONCLUSÕES:**

A redução na CV, VC e VM foi observada durante o período pós-operatório em pacientes submetidos a clipagem de aneurisma ou ressecção de tumor. A redução na CV e no VC foi maior em pacientes submetidos a craniotomia por aneurisma com tempo cirúrgico mais longo.

## INTRODUCTION

Changes in pulmonary function commonly occur after general surgery.^1–7^ These changes include decreased lung volume ranging from 25% to 60%,^8^ decreased tidal volume (TV), increased respiratory frequency,^9^ gas exchange changes with decreased partial oxygen pressure^10,11^ and decreased mucociliary clearance.^9^ These changes occur more intensely in surgery involving the abdominal and thoracic cavity, and they contribute towards the development of pulmonary complications.^1,12–14^

Craniotomy is considered to be peripheral surgery. It is commonly said that surgery that does not involve incision of the abdominal or thoracic cavities has fewer effects on postoperative pulmonary function, with lower incidence of pulmonary complications.^2,9,15^ As far as we know, there are no studies reporting on postoperative lung volume follow-up among patients who underwent craniotomy.

## OBJECTIVE

The aim of this study was to prospectively study a group of patients who underwent elective craniotomy for tumor resection or aneurysm clipping, in order to analyze the changes in pulmonary function and possible associations between the changes in vital capacity, tidal volume, minute volume and respiratory rate, neurological diseases and surgery duration.

## METHODS

This study was conducted among patients who underwent elective craniotomy because of a tumor or arterial aneurysm, under general anesthesia. This study was approved by the Ethics Committee for Human Research of Universidade Federal de São Paulo — Escola Paulista de Medicina (Unifesp-EPM).

The study patients maintained spontaneous ventilation and a consciousness level of P2 R1 D1 V1, according to the Jouvet Scale,^16^ during the postoperative period. This consciousness level is considered to be within normal limits. The Jouvet Coma Scale (JCS) was used in this study instead of the Glasgow Coma Scale (GCS) because JCS shows better sensitivity to consciousness levels close to normal, while GCS is more sensitive for intense loss of consciousness.^17^

Forty patients were evaluated and 26 of them were included in the study. Among the 14 patients excluded, one suffered brain death immediately after surgery, seven presented diminished consciousness level after the operation, which prevented the ventilation measurements, and six remained on mechanical ventilation for more than 24 hours.

All the patients included were evaluated and followed up by the same investigator, who knew the type of surgery that the patients were undergoing. The evaluation was done according to a standardized evaluation chart consisting of clinical history, physical examination and ventilation measurements using an Ohmeda ventilometer (model RM 121, Ohmeda, Japan). The clinical history was obtained in order to determine the presence of respiratory symptoms, pulmonary disease and smoking habit at the time of surgery.

The ventilation measurements were made before the operation and from the first to the fourth postoperative day, and included vital capacity (VC), tidal volume (TV), minute volume (VE) and respiratory rate (RR). These measurements were made with the patient positioned in dorsal decubitus at 45 degrees, a position in which patients are usually kept during the postoperative period.

The patients were followed up daily, and physical examinations with ventilation measurements were performed. Respiratory physiotherapy involving bronchial hygiene maneuvers and pulmonary expansion exercises was administered from the immediate postoperative period until discharge or the occurrence of death during hospitalization. Patients were advised to start walking again at an early stage. There were no contraindications for physiotherapy among these patients. Moreover, they presented normal consciousness levels and were able to undergo the therapy.

The patients were medicated with analgesics before and after the operation. This was done step by step, based on each patient's intensity of pain, subjectively. The patients were initially medicated with an anti-inflammatory drug (tenoxicam); if they complained of pain, the medication sequence continued with amitriptyline, then carbamazepine, chlorpromazine and finally an opioid (tramadol). Before ventilometer measurements, the patients were asked about pain and, if necessary, were medicated. All medications were given orally, and the doses of analgesic were enough to keep patients comfortable.

The data were statistically analyzed by analysis of variance for repeated measurements, Friedman rank analysis of variance and the Mann-Whitney test, with the level of significance set at 5% in all cases.

## RESULTS

The demographic characteristics of the 26 patients are shown in Table 1. None of the patients studied presented respiratory symptoms or pulmonary disease at the time of surgery, based on the absence of respiratory symptoms.

**Table 1.** Demographic characteristics of the 26 patients included in the study

Characteristics	Patients
**Gender**	
Male	13
Female	13
**Mean age (years)**	
Male (mean ± SD)	40 ± 17
Female (mean ± SD)	37 ± 12
**Smokers**	6
Pack-years (mean ± SD)	19 ± 13
**Former smokers**	8
**Pack-years (mean ± SD)**	19 ± 22
**Non-smokers**	12
**Mean surgery duration (mean ± SD in minutes)**	274 ± 108
**Orotracheal intubation (mean ± SD in hours)**	16 ± 5

Pack-years = number of cigarettes per day divided by 20 and multiplied by the number of years for which the individual has been smoking; SD = standard deviation.

**Figure 1:**
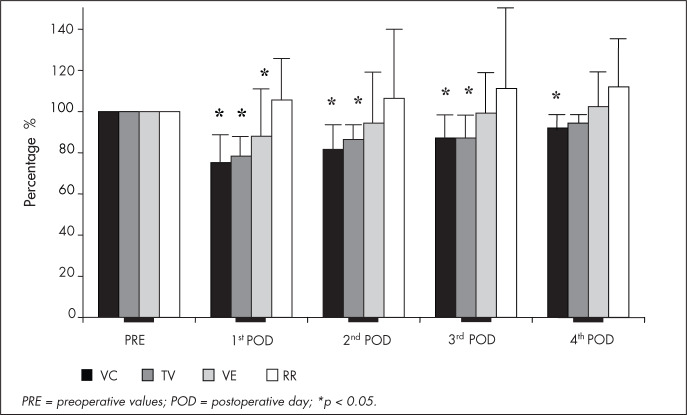
Comparison of vital capacity (VC), tidal volume (TV), minute volume (VE) and respiratory rate (RR) values on the preoperative period (PRE) and on the first, second, third and fourth postoperative days (POD) for 26 patients who underwent craniotomy because of aneurysms or tumors, in comparison with preoperative values. Values are reported as $\Delta \%=\frac{\text{postoperative}-\text{preoperative }\times 100}{\text{preoperative}}$

All postoperative VC measurements showed significant decreases (p < 0.05) in relation to preoperative values: decreases of 25 ± 15%, 19 ± 14%, 13 ± 11% and 8 ± 8% on the first to fourth postoperative days respectively. There were significantly decreased TV measurements of 22 ± 11% on the first postoperative day, 14 ± 8% on the second and 13 ± 10% on the third p < 0.05), whereas the TV decrease of 6 ± 15% on the fourth postoperative day was non-significant (p > 0.05). VE was significantly lower (p < 0.05) only on the first postoperative day (12% ± 24 %). RR did not differ significantly from before to after the operation, although it was 5 ± 19%, 6 ± 33%, 11 ± 39% and 12 ± 33% higher on the first to fourth postoperative days respectively (Figure 1).

To determine whether duration and type of surgery were related to the decreases in VC, TV and VE, the patients were divided according to the median duration of surgery (four hours) and the neurological diseases that were the reason for the surgery (aneurysm or tumor). Thus, two groups were formed: the first with surgery duration exceeding four hours and consisting of six patients with a diagnosis of aneurysm and six patients with a diagnosis of tumor; and the second with surgery duration of four hours or less and consisting of five patients with a diagnosis of aneurysm and nine patients with a diagnosis of tumor.

**Figure 2:**
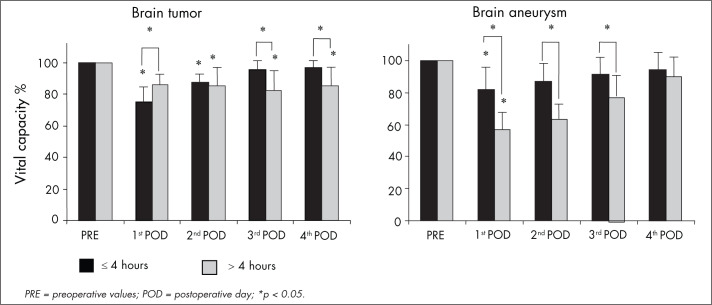
Comparison of vital capacity before surgery (PRE) and on the first, second, third and fourth postoperative days (POD) for 11 patients who underwent craniotomy because of brain aneurysms and 15 patients who underwent craniotomy because of brain tumors, with surgery duration greater than or less than four hours.

The patients who underwent craniotomy because of a tumor whose surgery lasted for more than four hours, showed decreased VC (p < 0.05) on the second, third and fourth postoperative days (15 ± 12%, 18 ± 12% and 15 ± 9% respectively) while the patients who underwent craniotomy because of a tumor whose surgery lasted for less than four hours presented decreased VC (p < 0.05) on the first and second postoperative days (25 ± 9% and 13 ± 5% respectively). The patients who underwent aneurysm clipping whose surgery lasted for more than four hours showed a greater decrease in VC (p < 0.05) on the first, second and third postoperative days (43 ± 13 %, 37 ± 12% and 22 ± 11% respectively) than did the patients with shorter surgery duration, with decreases of 18 ± 14%, 13 ± 10% and 9 ± 8%, respectively (Figure 2).

**Figure 3:**
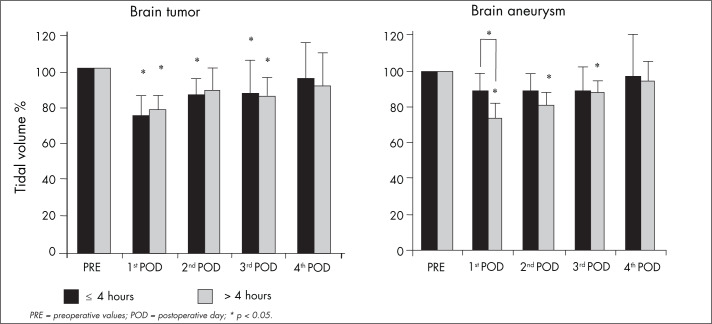
Comparison of tidal volume before surgery (PRE) on the first, second, third and fourth postoperative days (POD) for 11 patients who underwent craniotomy because of brain aneurysms and 15 patients who underwent craniotomy because of brain tumors, with surgery duration greater than or less than four hours.

There was no relationship between surgery duration and reduction in TV among the patients who underwent craniotomy because of a tumor. The patients who underwent aneurysm clipping were found to present significantly decreased TV (26 ± 7%) on the first postoperative day (p < 0.05) when the surgery duration was more than four hours, in comparison with patients whose surgery duration was less than four hours (11 ± 7%) (Figure 3).

**Figure 4:**
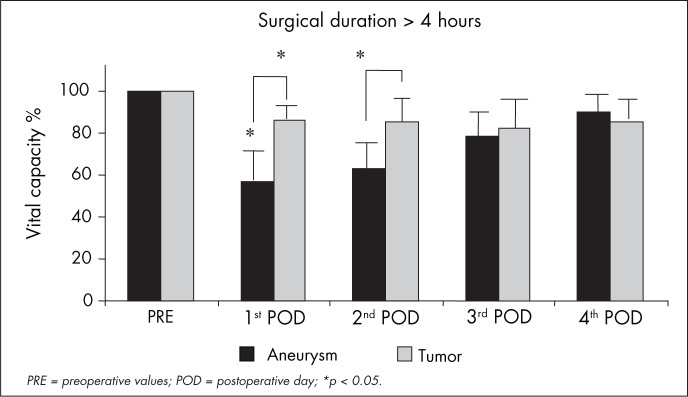
Comparison of vital capacity before surgery (PRE) and on the first, second, third and fourth postoperative days (POD) for 14 patients who underwent craniotomy because of brain tumors (n = 6) and brain aneurysms (n = 6), with surgery duration greater than four hours.

With regard to VC after surgery lasting more than four hours, patients with aneurysm clipping showed a significantly greater reduction on the first and second postoperative days (43 ± 13% and 37 ± 12%) than did the patients who underwent craniotomy for tumor resection (14 ± 6% and 15 ± 12%) (p < 0.05). The reduction in VC observed after surgery for aneurysm clipping did not differ from what was observed for tumor resection when the surgery duration was less than four hours (Figure 4).

Comparing the TV values for surgery lasting less than four hours, a more significant reduction (p < 0.05) was observed on the first postoperative day in patients who underwent tumor resection (25 ± 11%) than in patients who underwent aneurysm clipping (11 ± 7%).

No changes in VE were detected between patients undergoing tumor resection or aneurysm clipping with the same surgery duration.

No significant difference in respiratory rate was observed between the preoperative and postoperative evaluations when the 26 patients were divided according to duration and type of surgery. Likewise, no significant difference in respiratory rate was observed between patients undergoing aneurysm clipping or tumor resection with the same surgery duration. There was also no difference in pulmonary function between smokers and non-smokers.

## DISCUSSION

The present study demonstrated that there were changes in pulmonary function in patients who underwent craniotomy because of a tumor or aneurysm. These changes consisted of significant decreases (p < 0.05) in VC (25%), TV (22%) and VE (12%) and a non-significant increase in respiratory frequency (5%) on the first postoperative day.

The changes in VC and TV observed in the present study were similar to those observed in patients undergoing lower abdominal surgery^4,5^ and open fundoplication surgery.^18^ In these situations, the fall in VC results from a combination of factors, including the action of the anesthetics, diaphragmatic paresis, immobility in bed and postoperative pain.^9^ On the other hand, different results have been found among patients undergoing thoracic^19,20^ and upper abdominal surgery.^1^

The duration of the changes in VC and TV that we saw was also similar to changes that have been observed in lower abdominal surgery.^4,5,21^ We found that, although VC and TV were still 8% and 6%, respectively, below preoperative values on the fourth postoperative day, there was a clear trend towards returning to these preoperative values.

The reduction in volumes among patients undergoing craniotomy may be explained by the longer surgery duration required for these cases than for other types of peripheral surgery, since theoretically there is no diaphragmatic alteration or limitation of chest movements or of the abdominal cavity during respiration after this type of surgery.

It is possible that anesthesia played some role in determining the changes in pulmonary function observed. It is known that anesthetic agents and techniques not only boost the depression of the respiratory center but also interfere with intracranial pressure, since they lead to changes in cerebral blood flow, cerebrospinal fluid dynamics and brain edema production. This increase in intracranial pressure causes a reduction in cerebral perfusion pressure, thereby generating ischemic areas and consequently changes in brain metabolism and respiratory patterns.^22–24^

Among the patients who underwent aneurysm clipping, the reduction in VC was greater (p < 0.05) than among those whose surgery duration exceeded four hours (43%) compared to the reduction observed in patients with a surgery duration of four hours or less (19%). This result permits us to state that surgery duration was one of the factors that induced the greatest reduction in VC in this group of patients.

However, in the patients who underwent craniotomy for tumor resection, the decrease in VC was inversely correlated with surgery duration, contrary to what was expected. Patients with surgery duration of four hours or more presented a 14% decrease in VC, while those with surgery duration of four hours or less presented a 25% decrease in VC (p < 0.05).

This unequal influence of surgery duration on the results from craniotomy because of aneurysms or tumors may perhaps be explained by the difference in the specific surgical procedures performed in the two groups of patients. Aneurysm clipping is performed using microsurgical techniques and usually 85% of the aneurysms are located in the large base vessels, which leads to deeper manipulation of the encephalic parenchyma than does the removal of expansive processes.^25^ Cerebral vasospasm may occur in 70% of patients during the first two weeks after bleeding caused by ruptured aneurysms.^26^ Thus, vasospasms may or may not be symptomatic and they are responsible for a large proportion of the morbidity and mortality due to ruptured aneurysms.^25,26^ The reduction in blood flow following vasospasm may lead to neural metabolic changes and consequently to changes in respiratory patterns. However, in the present study all the operations for aneurysm clipping were performed after the critical period of vasospasm formation, which rules out any effect from this complication in relation to triggering changes in respiratory patterns.

Manipulation of the encephalic parenchyma to remove an expansive intracranial process leads to acidosis of cerebrospinal fluid pH, a parameter that demonstrates the occurrence of changes in neuronal metabolism.^27^ These changes in neuronal metabolism occurring postoperatively in patients undergoing craniotomy because of tumors have been reported by Ducker and Redding^28^ to be one of the causes of changes in respiratory patterns. This may partially explain the changes in pulmonary function detected postoperatively in patients who underwent craniotomy because of tumors.

The present observation of changes in pulmonary function during the postoperative period in patients who underwent craniotomy because of aneurysms or tumors indicates the potential of this type of surgery for triggering the onset of pulmonary complications during the postoperative period. The occurrence of changes in pulmonary function during this period in such patients, together with the severity of the disease itself, increases the possibilities for pulmonary complications.

Indeed, due to the severity of the disease, and regardless of the type of surgery performed, patients may present changes in respiratory pattern, reduction of consciousness level, modification of the bacterial flora and neurogenic changes that favor the development of pulmonary complications.^5,21,29–33^

Thus, in view of the susceptibility of this group of patients to the development of postoperative respiratory complications, the indication of physiotherapy before and after the operation is justified, since several studies have confirmed the efficiency of such treatment for reducing pulmonary complications in patients undergoing abdominal surgery.^34–37^

## CONCLUSIONS

In summary, the present study showed that, among patients undergoing craniotomy, VC and TV are lowered at least up to the fourth postoperative day and VE is lowered up to the second postoperative day. Surgery duration of more than four hours for patients undergoing craniotomy because of aneurysms caused a greater decrease in VC and TV than seen among patients whose surgery lasted for less than four hours. The reduction in pulmonary volumes was greater for patients undergoing craniotomy because of aneurysms with surgery duration exceeding four hours, while there was no difference between tumor and aneurysm for surgery duration of less than four hours.

## References

[1] Dureuil B, Cantineau JP, Desmonts JM (1987). Effects of upper or lower abdominal surgery on diaphragmatic function. Br J Anaesth.

[2] Ozdilekcan C, Songur N, Berktas BM, Dinç M, Uçgül E, Ok U (2004). Risk factors associated with postoperative pulmonary complications following oncological surgery. Tuberk Toraks.

[3] Craig DB (1981). Postoperative recovery of pulmonary function. Anesth Analg.

[4] Jackson CV (1988). Preoperative pulmonary evaluation. Arch Intern Med.

[5] Smetana GW (1999). Preoperative pulmonary evaluation. N Engl J Med.

[6] Arabaci U, Akdur H, Yigit Z (2003). Effects of smoking on pulmonary functions and arterial blood gases following coronary artery surgery in Turkish patients. Jpn Heart J.

[7] Bigler DR (2003). Aendringer i lungefunktionen ved anaestesi og thoraxkirurgi. [Lung function changes during anesthesia and thoracic surgery]. Ugeskr Laeger.

[8] Latimer RG, Dickman M, Day WC, Gunn ML, Schmidt CD (1971). Ventilatory patterns and pulmonary complications after upper abdominal surgery determined by preoperative and postoperative computerized spirometry and blood gas analysis. Am J Surg.

[9] Sprung J, Gajic O, Warner DO (2006). Review article: Age related alterations in respiratory function – anesthetic considerations: [Article de synthese: Les modifications de fonction respiratoire liees a l'age - considerations anesthesiques]. Can J Anaesth.

[10] Aldren CP, Barr LC, Leach RD (1991). Hypoxaemia and postoperative pulmonary complications. Br J Surg.

[11] Reeder MK, Goldman MD, Loh L (1992). Postoperative hypoxaemia after major abdominal vascular surgery. Br J Anaesth.

[12] Schauer PR, Luna J, Ghiatas AA, Glen ME, Warren JM, Sirinek KR (1993). Pulmonary function after laparoscopic cholecystectomy. Surgery.

[13] Rothen HU, Neumann P, Berglund JE, Valtysson J, Magnusson A, Hedenstierna G (1999). Dynamics of re-expansion of a atelectasis during general anaesthesia. Br J Anaesth.

[14] Claxton BA, Morgan P, McKeague H, Mulpur A, Berridge J (2003). Alveolar recruitment strategy improves arterial oxygenation after cardiopulmonary bypass. Anaesthesia.

[15] Bryant LR, Preston D, Houck G, Mobin-Uddin K, Trinkle JK, Griffen WO (1972). Lung perfusion scanning for estimation of postoperative pulmonary function. Arch Surg.

[16] Jouvet M (1964). [Neurophysiological clinical study of disorders of consciousness]. Acta Neurochir (Wien).

[17] Muniz EC, Thomaz MC, Kubota MY, Cianci L, de Sousa RM (1997). Utilização da escala de coma de Glasgow e escala de coma de Jouvet para avaliação do nível de consciência. [Use of the Glasgow Coma Scale and the Jouvet Coma Scale to evaluate the level of consciousness]. Rev Esc Enferm USP.

[18] Olsén MF, Josefson K, Dalenbäck J, Lundell L, Lönroth H (1997). Respiratory function after laparoscopic and open fundoplication. Eur J Surg.

[19] Guizilini S, Gomes WJ, Faresin SM (2004). Efeitos do local de inserção do dreno pleural na função pulmonar no pós-operatório de cirurgia de revascularização do miocárdio. [Effects of the pleural drain site on the pulmonary function after coronary artery bypass graf]. Rev Bras Cir Cardiovasc.

[20] Vargas FS, Terra M, Hueb W, Teixeira LR, Cukier A, Light RW (1997). Pulmonary function after coronary artery bypass surgery. Respir Med.

[21] Pereira ED, Fernandes AL, da Silva Anção M, de Araúja Pereres C, Atallah AN, Faresin SM (1999). Prospective assessment of the risk of postoperative pulmonary complications in patients submitted to upper abdominal surgery. Sao Paulo Med J.

[22] Michenfelder JD, Cucchiara RF, Michenfelder JD (1990). Cerebral blood flow and metabolism. Clinical neuroanesthesia.

[23] Ducker TB, Simmons RL (1968). Increased intracranial pressure and pulmonary edema. 2. The hemodynamic response of dogs and monkeys to increased intracranial pressure. J Neurosurg.

[24] Artru AA, Nugent M, Michenfelder LD (1982). Enflurane causes prolonged and reversible increase in the rate of CSF production in the dog. Anesthesiology.

[25] Greenberg MS, Greenberg MS (1997). SAH and aneurysms. Handbook of neurosurgery.

[26] Kassell NF, Sasaki T, Colohan AR, Nazar G (1985). Cerebral vasospasm following aneurysmal subarachnoid hemorrhage. Stroke.

[27] Zupping R (1972). Cerebral metabolism in patients with intracranial tumors. J. Neurosurg.

[28] Ducker TB, Redding JS (1976). Pulmonary complications in neurosurgery. Clin Neurosurg.

[29] Kocabas A, Kara K, Ozgur G, Sonmez H, Burgut R (1996). Value of preoperative spirometry to predict postoperative pulmonary complications. Respir Med.

[30] Romig DA, Voth DW, Liu C, Brackett CE (1973). Bacterial flora and infection in patients with brain injury. J Neurosurg.

[31] Akça O, Koltka K, Uzel S (2000). Risk factors for early-onset, ventilator-associated pneumonia in critical care patients: selected multiresistant versus nonresistant bacteria. Anesthesiology.

[32] Wauchob TD, Brooks RJ, Harrison KM (1984). Neurogenic pulmonary oedema. Anaesthesia.

[33] Hsieh AH, Bishop MJ, Kubilis PS, Newell DW, Pierson DJ (1992). Pneumonia following closed head injury. Am Rev Respir Dis.

[34] Chumillas S, Ponce JL, Delgado F, Viciano V, Mateu M (1998). Prevention of postoperative pulmonary complications through respiratory rehabilitation: a controlled clinical study. Arch Phys Med Rehabil.

[35] Morran CG, Finlay IG, Mathieson M, McKay AJ, Wilson N, McArdle CS (1983). Randomized controlled trial of physiotherapy for postoperative pulmonary complications. Br J Anaesth.

[36] Roukema JA, Carol EJ, Prins JG (1988). The prevention of pulmonary complications after upper abdominal surgery in patients with noncompromised pulmonary status. Arch Surg.

[37] Hall JC, Tarala RA, Tapper J, Hall JL (1996). Prevention of respiratory complications after abdominal surgery: a randomised clinical trial. BMJ.

